# Functional variant of the P2X7 receptor gene is associated with human papillomavirus-16 positive cervical squamous cell carcinoma

**DOI:** 10.18632/oncotarget.12636

**Published:** 2016-10-13

**Authors:** Yuh-Cheng Yang, Tzu-Yang Chang, Tze-Chien Chen, Wen-Shan Lin, Shih-Chuan Chang, Yann-Jinn Lee

**Affiliations:** ^1^ Department of Gynecology and Obstetrics, MacKay Memorial Hospital, Taipei City, Taiwan; ^2^ Department of Pediatric Endocrinology, MacKay Children's Hospital, Taipei City, Taiwan; ^3^ Department of Medical Research, MacKay Memorial Hospital, New Taipei City, Taiwan; ^4^ Department of Gynecology and Obstetrics, School of Medicine, College of Medicine, Taipei Medical University, Taipei City, Taiwan; ^5^ Department of Pediatrics, School of Medicine, College of Medicine, Taipei Medical University, Taipei City, Taiwan; ^6^ Institute of Biomedical Sciences, Mackay Medical College, New Taipei City, Taiwan; ^7^ Department of Medicine, Mackay Medical College, New Taipei City, Taiwan

**Keywords:** cervical cancer, HPV, immunity, P2RX7, polymorphism

## Abstract

Human papillomavirus (HPV) infection and the fate of HPV infected cervical epithelial cells are strictly associated with cervical cancer development. P2X7 receptor has been implicated in both the regulation of immune responses and apoptosis of cervical cancer cells. The study aims to investigate if polymorphisms in the *P2RX7* gene are associated with the risk of cervical cancer in Taiwanese women. *P2RX7 253 T/C*, *835 G/A*, and *1513 A/C* loss-of-function polymorphisms were genotyped in a hospital-based study of 507 women with cervical squamous cell carcinoma (CSCC) and 1619 age-matched healthy control women. The presence and genotypes of HPV in CSCC was determined. The frequency of *253 C/C* genotype was found to increase significantly in patients with HPV-16 positive CSCC compared with controls (odds ratio = 10.2, 95% confidence interval 1.39–87.8, *P_c_* = 0.03). No significant associations were found for other 2 polymorphisms. Analysis of haplotypes also revealed no significant differences among women with CSCC, those with HPV-16 positive CSCC and controls. In conclusion, inheritance of the *C/C* genotype at position 253 in the *P2RX7* gene may contribute to the risk of HPV-16 associated CSCC in Taiwanese women.

## INTRODUCTION

Cervical cancer is the fourth most common cancer in women worldwide. In Taiwan, cervical cancer also poses a major public health concern, with nearly 2700 women were diagnosed with cervical cancer each year [1]. Human papillomavirus (HPV) infection and the subsequent alterations in growth and differentiation of infected cervical epithelial cells are important in cervical cancer development [2]. However, the majority of women with HPV do not develop cervical cancer, suggesting that other environmental factors are required for the cancer to develop. In addition to the environmental factors, host immune system is essential in determining the outcome of HPV infection and progression of cervical cancer [3].

The P2X7 receptor is a homotrimeric ligand-gated cation channel characterized by two transmembrane domains, an extracellular loop, an intracellular N terminus, and a long C-terminal chain containing 240 residues [4, 5]. This receptor has been found on many cells throughout the body and is most highly expressed on immune cells [6]. ATP is the naturally occurring ligand for the P2X7 receptor and activation of the receptor by extracellular ATP causes the movement of Ca^2+^, Na^+^, and K^+^ across the plasma membrane [7]. P2X7 receptor activation has been demonstrated to regulate the immune response by processing and release of cytokines such as IL-1β, IL-2, and IL-18 [8, 9], activation and proliferation of T cells [10, 11], possible involvement in antigen presentation [12], inhibition of soluble and membrane-bound HLA-G production [13], and the shedding of L-selectin (CD62L) [14]. Furthermore, the immune function of P2X7 receptor has been consolidated by studies of mice lacking P2X7 receptors [15, 16].

The human P2X7 receptor gene (*P2RX7*) contains 13 exons and is mapped to the 12q24 chromosomal region [17]. Several single nucleotide polymorphisms (SNPs) identified in the coding region of *P2RX7* gene have been reported to affect receptor function [18]. Given the crucial role of P2X7 receptor in immune modulation, *P2RX7* SNPs have been studied in several types of cancers like breast cancer, chronic lymphocytic leukemia, nasopharyngeal carcinoma, and papillary thyroid cancer [18]. However, association between the *P2RX7* SNPs and cervical cancer has not been determined. We therefore performed an association study of 507 cervical squamous cell carcinoma (CSCC) cases and 1619 controls to test whether specific *P2RX7* SNPs are associated with susceptibility to CSCC.

## RESULTS

The detection rate for HPV DNA in 507 CSCC samples was 72.6%. The HPV type distributions were observed that HPV 16 was 65.2%, HPV 18 was 9.3%, and the remaining types were 25.5%.

The genotype and allele distributions of *P2RX7* SNPs in cases and controls were shown in Tables 1–3. The genotype frequencies of all 3 SNPs in the control group did not deviate significantly from Hardy-Weinberg equilibrium (*P* > 0.05). We found the genotypes of *253 T/C* SNP in controls differed significantly from CSCC patients (*P* = 0.01) (Table 1). However, the increased frequency of *C/C* genotype (OR = 6.43, 95% CI 1.02–50.6) in CSCC patients did not survive the Bonferroni correction (*P_c_* = 0.06). For the other 2 polymorphic sites examined on the *P2RX7* gene, no significant difference was found in relation to the risk of CSCC (Tables 2–3).

**Table 1:** Genotype and allele frequencies of the *P2RX7 253 T/C* polymorphism in controls and in women with CSCC and those with HPV-16 positive CSCC*

	Controls (N =1619)	CSCC (N = 507)	HPV-16 positive CSCC (N = 240)	CSCC		HPV-16 positive CSCC	
	n (%)	n (%)	n (%)	*P* value (χ)	OR (95% CI)	*P* value (χ)	OR (95% CI)
Genotype				0.01 (8.74)		0.002 (12.1)	
T/T	1507 (93.1)	479 (94.5)	227 (94.6)		1.27 (0.83-1.95)		1.30 (0.72-2.34)
T/C	110 (6.8)	24 (4.7)	10 (4.2)		0.68 (0.43-1.07)		0.60 (0.31-1.16)
C/C	2 (0.1)	4 (0.8)	3 (1.3)		6.43 (1.02-50.6)		10.2 (1.39-87.8)
Allele				0.58 (0.31)		0.83 (0.04)	
T	3124 (96.5)	982 (96.8)	464 (96.7)		1.12 (0.75-1.67)		1.06 (0.62-1.80)
C	114 (3.5)	32 (3.2)	16 (3.3)		0.89 (0.60-1.33)		0.94 (0.55-1.61)

*CSCC = cervical squamous cell carcinoma; HPV = human papillomavirus; OR = odds ratio; CI = confidence interval.

**Table 2:** Genotype and allele frequencies of the *P2RX7 835 G/A* polymorphism in controls and in women with CSCC and those with HPV-16 positive CSCC*

	Controls (N = 1619)	CSCC (N = 507)	HPV-16 positive CSCC (N = 240)	CSCC		HPV-16 positive CSCC	
	n (%)	n (%)	n (%)	*P* value (χ)	OR (95% CI)	*P* value (χ)	OR (95% CI)
Genotype				0.32 (2.28)		0.15 (3.75)	
G/G	449 (27.7)	131 (25.8)	54 (22.5)		0.91 (0.72-1.14)		0.76 (0.55-1.04)
G/A	824 (50.9)	252 (49.7)	137 (57.1)		0.95 (0.78-1.16)		1.28 (0.98-1.69)
A/A	346 (21.4)	124 (24.5)	49 (20.4)		1.19 (0.94-1.51)		0.94 (0.67-1.32)
Allele				0.17 (1.92)		0.38 (0.77)	
G	1722 (53.2)	514 (50.7)	245 (51.0)		0.91 (0.79-1.04)		0.92 (0.76-1.11)
A	1516 (46.8)	500 (49.3)	235 (49.0)		1.10 (0.96-1.27)		1.09 (0.90-1.32)

* CSCC = cervical squamous cell carcinoma; HPV = human papillomavirus; OR = odds ratio; CI = confidence interval.

**Table 3:** Genotype and allele frequencies of the *P2RX7 1513 A/C* polymorphism in controls and in women with CSCC and those with HPV-16 positive CSCC*

	Controls (N = 1619)	CSCC (N = 507)	HPV-16 positive CSCC (N = 240)	CSCC		HPV-16 positive CSCC	
	n (%)	n (%)	n (%)	*P* value (χ)	OR (95% CI)	*P* value (χ)	OR (95% CI)
Genotype				0.77 (0.54)		0.48 (1.47)	
A/A	944 (58.3)	304 (60.0)	146 (60.8)		1.07 (0.87-1.31)		1.11 (0.84-1.47)
A/C	578 (35.7)	172 (33.9)	84 (35.0)		0.92 (0.75-1.14)		0.97 (0.73-1.29)
C/C	97 (6.0)	31 (6.1)	10 (4.2)		1.02 (0.67-1.55)		0.68 (0.35-1.33)
Allele				0.62 (0.25)		0.30 (1.10)	
A	2466 (76.2)	780 (76.9)	376 (78.3)		1.04 (0.88-1.23)		1.13 (0.90-1.43)
C	772 (23.8)	234 (23.1)	104 (21.7)		0.96 (0.81-1.13)		0.88 (0.70-1.11)

* CSCC = cervical squamous cell carcinoma; HPV = human papillomavirus; OR = odds ratio; CI = confidence interval.

By stratification analysis based on HPV-16 positivity, the combined effect of HPV-16 infection and *P2RX7* polymorphisms on the risk of CSCC can be explored. We showed only *253 T/C* SNP of the HPV-16 infection subgroup differed significantly from control individuals (*P* = 0.002) (Table 1). The frequency of *C/C* genotype increased significantly in comparison with controls (OR = 10.2, 95% CI 1.39–87.8), and the significance remained after Bonferroni correction (*P_c_* = 0.03). Pairwise calculations of linkage disequilibrium between SNPs showed that there were strong linkage disequilibriums in controls (*D′* 0.86–1.00) and cases (*D′* 0.91–1.00).

The inferred haplotypes based on *253 T/C*, *835 G/A*, and *1513 A/C* SNPs were shown in Table 4. Globally, there were no significant differences in haplotype distribution between the CSCC or HPV-16 infection CSCC and control groups.

**Table 4:** Analysis of *P2RX7* haplotypes in controls and in women with CSCC and those with HPV-16 positive CSCC*

Haplotype	Controls (2N = 3238)	CSCC (2N = 1014)	HPV-16 positive CSCC (2N = 480)	CSCC		HPV-16 positive CSCC	
	2n (%)	2n (%)	2n (%)	*P* value (χ)	OR (95% CI)	*P* value (χ)	OR (95% CI)
TAA	1483 (45.8)	490 (48.3)	231 (48.1)	0.16 (1.98)	1.11 (0.96-1.27)	0.34 (0.91)	1.10 (0.91-1.33)
TGA	871 (26.9)	259 (25.5)	129 (26.9)	0.39 (0.73)	0.93 (0.79-1.10)	0.99 (0.01)	1.00 (0.80-1.24)
TGC	738 (22.8)	225 (22.2)	100 (20.8)	0.69 (0.16)	0.97 (0.82-1.14)	0.34 (0.92)	0.89 (0.70-1.13)
CGA	113 (3.5)	30 (3.0)	16 (3.3)	0.41 (0.67)	0.84 (0.56-1.27)	0.86 (0.03)	0.95 (0.56-1.62)

* Haplotype inferred using Haploview 4.2 program, based on the order of *253 T*/*C*, *835 G/A*, and *1513 A/C* polymorphisms.

CSCC = cervical squamous cell carcinoma; HPV = human papillomavirus; OR = odds ratio; CI = confidence interval.

*P* value for 4 haplotypes between CSCC patients and controls: *P* = 0.50 (χ = 2.37, 3 df).

*P* value for 4 haplotypes between HPV-16 positive CSCC patients and controls: *P* = 0.75 (χ = 1.23, 3 df).

## DISCUSSION

The current study explored association between specific functional SNPs of the *P2RX7* gene and their haplotypes and CSCC susceptibility in Taiwanese population. Our findings revealed that *253 C/C* genotype frequency increased significantly in the subgroup of CSCC women infected with HPV-16 as compared with healthy controls. In addition, it is noteworthy that the frequency of homozygous risk allele of the *253 T/C* SNP was very rare in controls, with only two was homozygous in the 1619 cohort (Table 1). Analysis of haplotype distribution did not reveal significant differences among the groups tested. Our results imply that the *P2RX7* gene might involve in the HPV-16 positive CSCC development. This study does have limitations: a selection bias in the study of retrospective design and scarcity of screened SNPs. Therefore, a large-scale prospective study that investigates more SNPs of *P2RX7* gene is needed to confirm our findings.

A few studies have reported that P2X7 expression was found in both normal and cancer cervical tissues and associated with the growth of cervical cells. A study reported by Li et al. showed that both protein and mRNA levels of the P2X7 were significantly lower in the cervical cancer than in the normal tissues [19]. They also found the total P2X7 immunostaining across the epithelium correlated reciprocally with the severity of the dysplasia of ectocervix. The same research group further discovered a novel P2X7 isoform: an inactive 42-45 kDa truncated variant (P2X_7-j_) that is equally expressed in normal and cancer cervical cells [20]. The lower levels of the full-length P2X7 expressed in cancer cells make them more likely to form nonfunctional P2X7 oligomers with P2X_7-j_ and abrogated the P2X7-mediated apoptosis. Moreover, P2X7 activation by extracellular ATP induces lesser ratio of apoptosis in cancer cervical cells than in normal human ectocervical cells through the mitochondrial pathway [21]. These findings suggested that abnormal expression and function of the P2X7 receptor may result in the cervical carcinogenesis.

The *P2RX7* gene is highly polymorphic and a number of SNPs within the coding region have been reported to affect P2X7 receptor function. The *C* allele of *253 T/C* SNP changes valine to alanine at residue 76 (V76A) in the extracellular loop and produces a partial loss of P2X7 function [22]. Another SNP, *835 G/A*, resulting in a change of the amino acid arginine to histidine at residue 270 (R270H) was also found to have a reduced receptor function [23]. The *1513 A/C* polymorphism causes an amino acid change from glutamic acid to alanine at residue 496 (E496A) in the C-terminus of P2X7 receptor and abolishes the receptor function [24]. The allelic dosage of the *1513 C* polymorphism has been shown to associate linearly with the loss of P2X7 receptor function. There are other variants causing either loss-of-function such as *474 G/A*, *853 G/A*, *946 G/A*, *1096 C/G*, and *1729 T/A*; or gain-of-function such as *489 C/T*, *1068 G/A*, and *1405 A/G* [18]. Overall, these non-synonymous SNPs of *P2RX7* gene are clearly involved in the regulation of P2X7 receptor function.

What molecular mechanisms lie beneath the association between *P2RX7* gene and HPV-16 positive CSCC risk are not addressed in this study. Nevertheless, based on our findings, it is conceivable to reason that impaired receptor function caused by *253 T/C* polymorphism may lead to decreased immune cell function and apoptosis of cervical cancer cells when stimulated with exogenous ATP. This specific *P2RX7* variant is therefore possible to lead to failure in clearance of HPV infections and the consequential CSCC development. If this correlation can be supported by other studies, women with this genotype should undergo cervical screening more frequently or receive prophylactic HPV vaccine.

In conclusion, we showed for the first time that patients possessing the *253 C/C* genotype and harboring HPV-16 infection are more susceptible to CSCC in Taiwanese women. Future studies that explore the associations between other functional *P2RX7* SNPs and CSCC should aid in defining the precise role of *P2RX7* gene in CSCC pathogenesis.

## PATIENTS AND METHODS

### Study subjects

A hospital-based case-control study was conducted at the Mackay Memorial Hospital. Five hundred and seven Taiwanese women with pathologically proven CSCC (mean ± SD age at diagnosis: 54.5 ± 12.5 years) were enrolled in the study. Controls were 1619 women (mean ± SD age at sampling: 53.1 ± 13.3 years) selected randomly from a cervical cancer screening program. Exclusion criteria included abnormal Pap result, history of cervical neoplasia, skin or genital warts, immunocompromised conditions, other cancers, and previous operations on the uterine cervix. All the control subjects were frequency matched to the cases by age. The study was approved by the Institutional Review Board of Mackay Memorial Hospital, in accordance with the Helsinki Declaration, and all participants provided informed written consent for use of their surgical resections or cervical scrapings.

### DNA extraction

Formalin-fixed, paraffin-embedded tissue blocks from CSCC patients were sectioned and dewaxed, and genomic DNA was then extracted using the Qiagen DNeasy Tissue Kit (Qiagen, Valencia, CA) according to the manufacturer's protocol. Genomic DNA of controls was extracted from cervical scrapings using Qiagen DNA extraction kit.

### HPV detection and typing

HPV detection and genotyping were performed by polymerase chain reaction (PCR)-based amplification of a fragment of approximately 192 bp in the L1 region of the HPV genome with a pair of degenerate primers, GP6+/MY11 [25, 26]. The PCR product was then sequenced on an automated sequencer (ABI 377, Applied Biosystems, Foster City, CA) to determine the HPV genotype. Since stratifications based on HPV types in controls were not performed in this study, no HPV DNA testing was done for the control subjects.

### *P2RX7* genotyping

The *253 T/C* (rs17525809), *835 G/A* (rs7958311), and *1513 A/C* (rs3751143) SNPs were genotyped because of their potential functional significance. They were determined using the Pre-Developed TaqMan Allelic Discrimination Assay (Applied Biosystems, Foster City, CA). Briefly, PCR were carried out in a 96-well GeneAmp PCR System 9700 (Applied Biosystems) with mixes consisting of 10 ng of genomic DNA, 5 μl of TaqMan Universal PCR Master Mix, 0.5 μl of 20× Assay Mix, and ddH_2_O to a final volume of 10 μl. Thermal cycle conditions were as follows: denaturation at 95°C for 10 min, followed by 40 cycles of denaturation at 92°C for 15 sec, and annealing and extension at 60°C for 1 min. After PCR, the TaqMan assay plates were transferred to the ABI PRISM 7000 Sequence Detection System (Applied Biosystems) where the endpoint fluorescence intensity in each well of the plate was read. The allele specific fluorescence data from each plate were analyzed using the SDS v.1.1 software (Applied Biosystems) to automatically determine the genotype of each sample.

### Statistical analysis

Statistical differences in frequency between each genotype, allele, and haplotype of CSCC, HPV-16 positive CSCC, and control groups were compared using chi-square test with Yates’ correction or Fisher's exact test (when the number of subjects in a cell was <5). Odds ratio (OR) and 95% confidence interval (CI) were calculated to determine the magnitude of associations. The Hardy-Weinberg equilibrium was assessed for each SNP in the control group by chi-square analysis. The frequencies of *P2RX7* haplotypes as well as linkage disequilibrium between paired SNPs in controls and cases were estimated using the Haploview 4.2 program [27]. Haplotypes with a frequency <2% in controls or cases were excluded. The Bonferroni correction, *P_c_* = 1 - (1-*P*)^n^, was used for multiple comparisons where *P_c_* is the corrected *P* value, *P* the uncorrected value, and n the number of comparisons [28]. In this study, n is 2 for each genotype and allele for simultaneously testing genotype and allele frequencies [29] but no correction for testing the 3 SNPs because of significant linkage among them [30] and 4 for each of the 4 haplotypes [30]. A corrected *P* value (*P_c_*) of <0.05 (2-tailed) was considered statistically significant. Using the Quanto Ver. 1.1 software (Department of Preventive Medicine, University of Southern California, CA, USA), we designed the study to have >90% power at a 5% significance level to determine a genotype relative risk of 1.8 conferred by *P2RX7* variants with an estimated prevalence of 360/100,000 [31].
